# Leveraging a Publish/Subscribe Fog System to Provide Collision Warnings in Vehicular Networks †

**DOI:** 10.3390/s19183852

**Published:** 2019-09-06

**Authors:** Subhadeep Patra, Pietro Manzoni, Carlos T. Calafate, Willian Zamora, Juan-Carlos Cano

**Affiliations:** 1 Department of Computer Engineering, Universitat Politècnica de València, Camino de Vera S/N, 46022 Valencia, Spain; 2 Faculty of Computer Science, Universidad Laica Eloy Alfaro de Manabí, Av. Circunvalación—Vía a San Mateo, 130214 Manta-Manabí, Ecuador

**Keywords:** fog computing, IoT, ITS, vehicular network, MQTT, FCW, Android, smartphones

## Abstract

Fog computing, an extension of the Cloud Computing paradigm where routers themselves may provide the virtualisation infrastructure, aims at achieving fluidity when distributing in-network functions, in addition to allowing fast and scalable processing, and exchange of information. In this paper we present a fog computing architecture based on a “content island” which interconnects sets of “things” to exchange and process data among themselves or with other content islands. We then present a use case that focuses on a smartphone-based forward collision warning application for a connected vehicle scenario. This application makes use of the optical sensor of smartphones to estimate the distance between the device itself and other vehicles in its field of view. The vehicle travelling directly ahead is identified relying on the information from the GPS, camera, and inter-island communication. Warnings are generated at both content islands, if the driver does not maintain a predefined safe distance towards the vehicle ahead. Experiments performed with the application show that with the developed method, we are able to estimate the distance between vehicles, and the inter-island communication has a very low overhead, resulting in improved performance. On comparing our proposed solution based on edge/fog computing with a cloud-based api, it was observed that our solution outperformed the cloud-based api, thus making us optimistic of the utility of the proposed architecture.

## 1. Introduction

Vaquero et al. [1] define fog computing as *“a scenario where a huge number of heterogeneous (wireless and sometimes autonomous) ubiquitous and decentralised devices communicate and potentially cooperate among them and with the network to perform storage and processing tasks without the intervention of third parties. These tasks include basic network functions or new services and applications that run in a sandboxed environment. Users leasing part of their devices to host these services get incentives for doing so.”* Hence, the fog should have the capability to handle constant changes in the number of participating nodes, and each node must be able to act as a router for its neighbours. Attributes like geo-distribution, location awareness, low latency, heterogeneity, and support for mobility, as well as real-time interactions, make fog computing compatible with Vehicular Networks (VNs), where nodes participating in the network are mobile vehicles and Roadside Units (RSUs). Fog computing can be specially useful in areas related to infotainment, safety, traffic support, and analytics. The basic architecture for computing, storage and networking for the Internet of Things (IoT) was presented by Bonomi et al. [2], which included some information on cloud computing and fog computing, is one of the first works in this area. [3] lays emphasis on the new dimension that IoT adds to big data and analytics owing to the participation of a massive number of distributed fog nodes. Later, Vaquero et al. [1] came up with the definition of fog computing that has been presented previously, offering a more broad and integrative view of the advances of fog computing at several levels. This concept of fog was further expanded by studying more elaborate scenarios involving smart buildings and Software-defined networking (SDN) by Stojmenovic et al. [4,5]. It was also seen in a related study [6] that taking advantage of the mobile fog as a platform for applications could result in more efficient applications by introducing lower latencies. Furthermore, other researchers like Zhu et al. [7] and Aazam et al. [8] suggested methods to optimise web page rendering and a smarter communication model, respectively, to further improve the performance of fog-based applications.

A fog computing architecture based on the idea of interconnected *content islands* each of which packs together groups of *things*, is proposed here. These so called things present within each content island are able to exchange data among themselves and also process them. A scenario where such an architecture would be useful is a vehicular network where each participating node/vehicle could be considered a content island equipped with various sensors and data capture devices. Another example could be a patient acting as a content island, sharing data from the multiple sensors used to monitor his or her health. This proposal is based on the integration of the Message Queue Telemetry Transport (MQTT) [9] protocol, a publish/subscribe messaging system to provide data and computation sharing. In fact, MQTT is already being used in a wide range of applications including motion-activated street lights [10] to save energy, pollution monitoring [11], remotely controlled robots [12], measuring the impact of earthquakes [13], monitoring device health [14], next-generation video surveillance [15], and smart home automation systems [16]. In the context of VNs and Intelligent Transportation System (ITS), [17] evaluates the adoption of MQTT into the AUTomotive Open System ARchitecture (AUTOSAR) [18]. Also, MQTT has been employed by Madisa et al. [19] to design a traffic control system to minimise the response time of emergency vehicles. The solution consists of an Android application that could change traffic signals, and a cellular network was used for communication purposes. Another interesting application taking advantage of the MQTT protocol was proposed by Taghizadeh et al. [20], which helps in sharing energy between two electric vehicles. In [21], the authors have evaluated the use of the MQTT, Hypertext Transfer Protocol (HTTP) [22], and Constrained Application Protocol (CoAP) [23] for edge-based and cloud-based service provisioning in VNs. A collision detection system that informs the local authorities of such occurrences has been presented in [24]. Shaout el al. [25] developed a system to detect children left behind in vehicles, also making use of the MQTT protocol.

We have validated the proposed architecture based on content islands using a real application that makes use of the optical sensor available in smartphones for detecting occasions when users do not maintain an established safe distance with the vehicle ahead. It requires the use of an Android device equipped with at least a back camera and a Global Positioning System (GPS) interface. The device is mounted on the dashboard of the vehicle with the camera facing ahead. Captured images from the camera are processed to calculate the distance from the lens of the camera to the other vehicles appearing in the image. Note that warnings are generated only when the driver does not maintain a safe distance between his or her vehicle and the one travelling just ahead. Both academia and industry, taking advantage of the different available technologies, developed ways to anticipate collisions before they actually happen. Woll [26], leveraging radar technology, produced one of the earliest works related to this area. Later in 2002, taking advantage of beacons, Miller et al. [27] designed a more advanced solution that could detect possible collisions without the need for the vehicles to be in actual line of sight. In the same year, Srinivasa [28] proposed a vision-based approach for the same purpose. This solution was further improved by combining data from a forward-looking camera and a radar, and it was presented in [29] a year later. Other approaches that involve the use of a camera are [30,31,32]. A couple of works that combine vision and radar-based approaches, like [33,34], resulted from Volvo’s [35] research on collision avoidance and automatic braking. Reference [36] is another application that uses a vision-based approach for the same purpose. Later, Chang et al. [37] studied the fusion of vision and GPS data for collision warning as GPS technology gained popularity. Misener et al. [38] proposed a solution where positional information of vehicles is exchanged to provide forward collision warning, assistance at intersections, detection of blind-spots, and aid during lane changes. Another similar approach based on the use of GPS and motion sensors is described in [39]. Fusion of data from the Light Detection and Ranging (LiDAR) and other on-board sensors available in vehicles was used in the solution proposed by Lei et al. [40].

The novelty of our approach is the adoption of readily available and cost-effective technologies in the form of smartphones, to develop a fog computing architecture for vehicular networks that can be used to develop a wide range of ITS applications. The rest of the paper is organised as follows: Section 2 describes the overall architecture of our proposal, and Section 3 dives deep into the functional details architecture taking into consideration a Forward Collision Warning (FCW) application. In the following section (Section 4), to prove the utility of the proposed architecture, we present some results from experiments performed with the FCW application that takes advantage of this architecture. In Section 5, which concludes this paper, we provide a summary of inferences drawn from the experimental results, and also discuss possible future research directions related to this area.

## 2. Proposed Architecture

This work derives inspiration from Gelernter [41] and his pioneering work named Linda, a distributed programming language. Linda was designed for system programming in distributed settings. The novelty of this programming language lies in the manner it handles interprocess communication. The nomenclature used in Linda is as follows: individual processes are referred to as workers, and interprocess messages are known as tuples. Thus, tuples are independent named entities that exist in a shared computation space called tuple space, that can be accessed by other processes/workers. Linda supports four types of operations that workers may make use of to manipulate tuples: *in* which is used to atomically read and remove a tuple from a tuple space; *rd* operation, on the other hand, allows non-destructive reading of a tuple space; *out* operation is used to generate and write a tuple; and finally *eval* is used to create new processes to evaluate tuples, and write the result obtained in the tuple space. Since our proposed architecture is based on the use of the MQTT protocol, thus a message which is associated to a defined topic can be considered a tuple. The supported operations in our case would simply be: *rd*, and *out* operations. A node subscribed to a particular topic in our case, could be considered to be using *rd* operation. On the other hand, publishing a message would be equivalent to a Linda *out* operation. These two basic operations, namely, *rd* and *out* could be used to provide more complex operations like *eval*. An *eval* operation can be considered a combination of one *rd* and two *out* operations. This means, a node can be subscribed to a topic (*rd* operation), receive a published message (*out* operation) from another node, manipulate or process the data received, and finally publish (*out* operation) the obtained result.

Once we are familiar with the reference model, we move on to define a content island. Content islands are a set of interconnected nodes or things which are capable of processing, sensing and exchanging data among themselves using a publish/subscribe system. Each content island contains an island core that handles flow of information within an island, as well as between different content islands. A message, in this context, can either contain data or an action trigger. A data message, as the name suggests, contains a set of values or information generated at a node within a content island. An example of contents of such data messages could include information related to the nitrogen and phosphorus content in soil. An action trigger on the other hand, could be a request originating from one node soliciting the other to process a dataset, turn off a device, or even complex operations like to click a photo and publish it online.

For our implementation of the proposed architecture, we have used the MQTT protocol to provide the required publish/subscribe-based communication. One of reasons for choosing MQTT is the fact that MQTT Application Program Interface (API) libraries are lightweight, they are optimised for the constrained devices and they are able to function even with low-bandwidth, high-latency or unreliable networks. These libraries do not have steep hardware or software requirements, making them compatible with even very simple devices.

**Figure 1:**
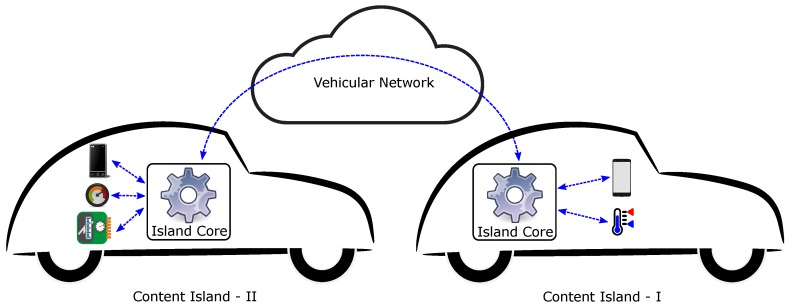
Concept of the island core.

Figure 1 shows each vehicle as a content island, where the island core is connected to the various sensors and devices present in the vehicle. Smartphones have grown in popularity in recent years, and almost everyone carries one in their pockets. It is highly probable that one or more smartphones may be present in the vehicles. Thus, content islands can also take advantage of the on-board sensors available to smartphones for monitoring different parameters. The island core receives data from the various devices/sensors present within a content island, and may perform some processing before sharing the received information with other devices present in the same content island, or with other content islands with which it is able to communicate. The hardware setup of the island core consists of two USB WiFi adaptors, both connected to a pocket-sized RaspberryPi [42]. One of the WiFi adaptors serves as an access point for devices within the vehicle, and the other one is configured to work in the ad hoc mode to facilitate direct communication with other content islands.

## 3. An Application to Test the Architecture

The application that is used to test the proposed architecture is a FCW application that has been specifically designed for highways with two lanes, where vehicles are moving in either direction, but only one lane is assigned per direction of movement. It aims to prevent collision by warning drivers when they are too close to the vehicle directly ahead that is on the same lane and moving in the same direction. Warnings are displayed at both the vehicle ahead and the one behind. Thus, we have to estimate the distance between the two vehicles for this purpose. In particular, we take advantage of the back camera of the dashboard-mounted smartphone present in the vehicle behind, acquiring images and estimating the distance from the dimensions of the identified license plate in the images. The processing of the images and calculation of the distance take place at the smartphone level. The distance between the vehicles can be estimated from the size of the license plate in images, as the dimensions of an actual physical license plate are fixed depending on the geographical region. If the estimated distance is less than the a predefined safe distance, warnings are generated on the smartphone, and these warning messages are also propagated to the content island ahead making use of the communication capabilities of the island core. This way, the warning message also reaches the vehicle ahead, and the driver in the vehicle ahead is also made aware of it.

Let us take a look at Figure 1 again. In the figure, we observe two vehicles travelling in the same direction, and on the same lane. We assume that both vehicles are using the discussed setup, consisting of a dashboard-mounted smartphone to be able to take advantage of our FCW application. The vehicle ahead has been named *Content Island-I*, while the vehicle following it is the *Content Island-II*. In this example, the smartphone camera in *Content Island-II* is used to continuously capture images, which are analysed to identify license plates within them. The distance between the two vehicles, namely, *Content Island-I* and *Content Island-II*, is estimated from the size of the plate in the image, coupled with the fact that license plates have a fixed physical dimension according to the geographical area. If this estimated distance is less than a defined safe distance, warnings are generated onscreen in the smartphone that is a part of *Content Island-II*. This warning is published to the island core of *Content Island-II*, which is then forwarded to the island core of *Content Island-I*. The warning message is received by the subscriber smartphone running our application, present in the *Content Island-I*, where a notification is displayed.

Next, we are going to present the software components of the island core that helps our application provide the desired FCW functionality. The island core comprises four functional elements: the MQTT broker, the Virtual Processing Client (VPC), the MQTT bridge, and the broadcast daemon.

**Figure 2:**
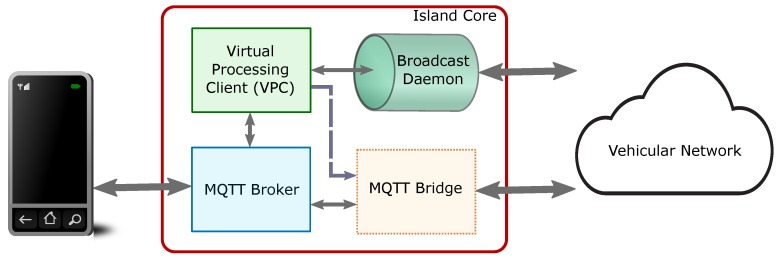
Functional components of the Island Core used by the FCW application.

Figure 2 depicts the different functional components. The MQTT broker is the element to which all devices and sensors present inside a content island are connected. These devices, also known as clients, connect to the broker and may publish information which is delivered to other clients that have subscribed to similar types of information. A VPC is a special client that is present as a part of the island core to control the flow of information for our application. The broadcast daemon is a service that is used by the VPC to send and receive broadcast message from other content islands, which is specially useful for neighbour discovery. Note that the broadcast daemon is the only component that does not use MQTT, but relies on the User Datagram Protocol (UDP) [43] for sending and receiving messages. Once neighbour discovery is complete, and if required, direct communication with another content island can be established. For this purpose, VPC sets up the MQTT bridge which helps to join two content islands for data exchange.

The functioning of our application can be explained using three steps:

1. **Neighbour discovery**: In this step, every smartphone running our application publishes an advertisement message to the island core. This message consists of an identifier, the actual license plate number of the vehicle within which the device is present, and the motion vector of the vehicle. At the same time, a device with our application running also subscribes to similar messages used for neighbour discovery. The island core, upon receiving such a message from a smartphone, appends its Internet Protocol (IP) address to the information, and shares it with other neighbouring islands. When a neighbouring island receives an advertisement message, it is forwarded to the device within that island and running our application. A list of suitable neighbours is prepared consisting of only those neighbours that are travelling in the same direction. Since the application has been designed to be used in scenarios involving two-laned roads with one lane per direction of traffic, we are left with neighbours travelling in the same direction that may be located ahead or behind the current vehicle.
2. **Distance estimation**: We rely on image processing for the estimation of distance between the two vehicles. Images captured continuously using the back camera of the dashboard mounted smartphone are analysed to recognise license plates appearing in them. Since the camera of the device faces the windshield, images contain vehicles present ahead, but that may be travelling in different directions. Comparing the identified plates with the entries in the neighbour list created in the previous step, which contains information regarding other vehicles travelling in the same direction, allows us to identify which vehicle is directly ahead. The size of the plate appearing in the images makes it possible to estimate the distance between the vehicles, and based on this estimate a decision about whether to generate a warning is made.
3. **Warning generation**: If the distance between the license plate of the vehicle travelling ahead and the camera of the device present in the vehicle behind is less than a defined safe distance, the drivers of both the vehicles are alerted. A warning is displayed on the smartphone screen at the vehicle behind, and taking advantage of inter-island communication, the driver of the vehicle ahead is also notified. The IP address of the island core of the vehicle ahead is known as it was a part of advertisement messages being broadcasted by each vehicle or content island; this information would appear in the neighbour list prepared in the first step.

We will now look in detail at each of these three steps.

### 3.1. Neighbour Discovery

Let us assume that we have an island core up and running with the initial components: an MQTT broker, VPC, and a broadcast daemon that are fully functional. The MQTT bridge is not configured at the beginning, but it is setup later when needed. The MQTT broker is responsible for pushing the information out to those clients that have previously subscribed to a particular topic. VPC is the brain of the island core, controlling its behaviour. The VPC is initially subscribed to the topics: *upv/grc/fcw/vector* to receive location updates, and *upv/grc/fcw/distances* to get estimates related to the distance of the current vehicle to license plates appearing in the images captured. Messages with such topics come from the smartphone running our application present in the same content island. The broadcast daemon, as the name suggests, is used to exchange advertisement messages among content islands.

Now, when the smartphone application is launched, the user needs to setup an identification string that is unique per content island. We use the license plate number of the vehicle as the unique identification string. As soon as the application starts working, it begins behaving as an MQTT client, subscribing to warning messages with topic *upv/grc/fcw/warning*. Messages with this topic would be originating from the vehicle travelling directly behind, and they are generated upon discovering that the vehicle behind is too close to the current vehicle. After the application setup phase is complete, the device uses the smartphone GPS to collect location information periodically. Once five such GPS points have been accumulated, it uses regression to compensate for errors in the last five known location points, and a motion vector of the vehicle is constructed. This motion vector is calculated every time an update from the GPS is received before storing this information locally. The motion vector and the license plate number supplied by the user are published on the MQTT broker, making use of the topic *upv/grc/fcw/vector*. The VPC, which is subscribed to the same topic, receives the message.

The VPC, upon receiving the motion vector and the license plate number from the smartphone, appends a local timestamp and the IP address of the interface working in adhoc mode. A copy of this modified message is retained locally, before being forwarded to the broadcast daemon for disseminating it to all the one-hop neighbours of the current content island. Similarly, when such a broadcast message is received by the broadcast daemon from one of the neighbouring content islands, it is sent to the VPC for further processing. The VPC which maintains a cache of previously received broadcast messages, first makes sure that the received message is recent and updates the cache accordingly. Next, it compares the motion vector of the current vehicle and the sender vehicle, taking advantage of the information contained within the received advertisement message. The angle between the motion vectors of the two content islands or vehicles is measured. Only if this angle is less than 90${}^{\circ }$ are the two vehicles considered to be moving in the same direction, in which case an entry is added or updated in the list of suitable neighbours, which is also maintained by the VPC. Thus, a list of all the one hop neighbours that are travelling in the same direction, but may be present ahead or behind the current vehicle, is prepared.

**Figure 3:**
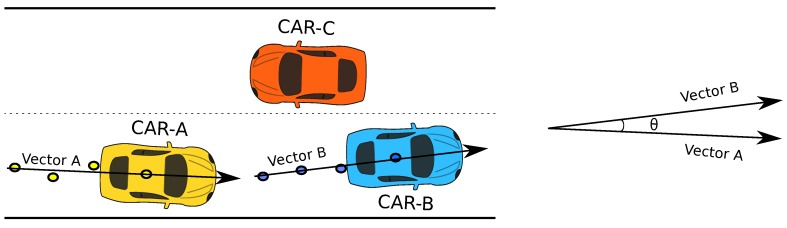
Suitable neighbour test.

Figure 3 explains the discussed tests performed to select the suitable neighbours for our application. As it can be seen from the example depicted in the figure, we have three vehicles: CAR-A, CAR-B and CAR-C. CAR-A and CAR-B are travelling in the same direction, while CAR-C is travelling in the opposite direction. Let us assume that our application is being used by all the vehicles in the example, meaning that each device running our application is aware of its current location. This location information, along with its previous four known GPS points, are used to construct the motion vector of each vehicle. The motion vectors are shared with one another through a broadcast message. Upon receiving the broadcast message, each vehicle compares its own motion vector with the received one. If the angle between vectors is less than 90${}^{\circ }$, the list of suitable neighbours is updated. This way, we are able to only select the neighbours that are travelling in the same direction. In this case, CAR-A only finds CAR-B to be travelling in the same direction, and vice versa. This way, they update their list of suitable neighbours appropriately.

### 3.2. Distance Estimation

While the application is listening for, or disseminating broadcast messages, the back camera of the smartphones is continuously used to capture the view ahead as seen by the driver of the vehicle. These images are processed to recognise all license plates appearing in the image. Before the images are processed, they are stored using the Joint Photographic Experts Group (JPEG) [44] encoding scheme. The first step in the process to recognise license plates within an image is to identify the regions within which license plates might exist. These regions within an image are called regions of interest, and the Local Binary Patterns (LBP) [45] algorithm is used to detect them. Once these regions of interest are identified, they are converted to black and white using algorithms designed by Wolf-Jolion [46] and Sauvola [47], before moving to the subsequent step. In the next step, we try to locate areas within the regions of interest which might contain characters. For this purpose, we begin looking for character-sized regions starting from the smallest first. Regions with groups of connected pixels or Binary Large OBject (BLOB) that are more or less similar to characters of a license plate are selected for further processing. This information, coupled with other data like the ratio of actual plate width and height depending on the geographical region, plays an important role when using the Hough transformation [48] to find the edges of the plate in the image. Any existing rotation or skew is taken care of by remapping the plate region. Next, the plate region is cleaned so that speckles and edges are not mistaken for characters. Also, a vertical histogram is used to detect gaps in characters, and all characters present are separated. Finally, each individual character is analysed, the degree of confidence is computed, and the best possible character combination is generated.

**Figure 4:**
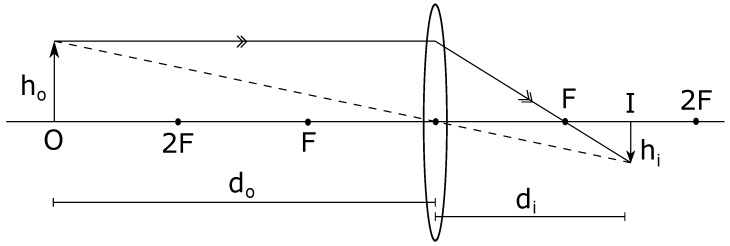
Refraction by convex lens when the object is beyond 2F.

Once the license plate has been identified, the distance is estimated using the dimensions of the identified plate region in the image. It is done in the following manner:

The general lens equation is:

(1) $$\frac{1}{f}=\frac{1}{d_{0}}+\frac{1}{d_{i}}$$

where *f* is the focal length of the camera lens, usually measured in millimetres. In case of our application, it is the focal length of the back camera of the android device that is used to capture the images. As can be seen in Figure 4, *f* is the distance between the center and the focal point (F) of the lens. The value of *f* is usually supplied by the manufacturer, but it can also be manually calculated very accurately by calibrating the lens. We rely on the manufacturer-supplied value in our application. In the same figure, we also observe that the distance of the lens from the object (O) being captured is $d_{o}$, while the distance from the lens to the image formed (I) is denoted by $d_{i}$.

Similarly,

$$\frac{d_{o}}{d_{i}}=\frac{h_{o}}{h_{i}}$$

(2) $$∴d_{i}=\frac{h_{i}d_{o}}{h_{o}}$$

In Equation (2), the height of the object (O) in the real world is denoted as $h_{o}$, and the height of the image (I) that represents the actual object (O) is $h_{i}$. Now, when talking about digital photographs, the entire view captured is referred to as an image, which may consist of one or more objects of interest. Since we are focused on these individual objects of interest that a photograph might contain, we will refer to them as the *object of interest in the image* in future to avoid confusion.

Next, we replace $d_{i}$ in Equation (1) with the new value acquired from Equation (2), and we get:

$$\frac{1}{f}=\frac{1}{d_{o}}+\frac{h_{o}}{h_{i}d_{o}}$$

(3) $$∴d_{o}=f\left(1+\frac{h_{o}}{h_{i}}\right)$$

In Equation (3), *f* is the manufacturer-supplied focal length of the lens, and $h_{o}$ is the height of the object in the real world. In our case, $h_{o}$ corresponds to the height of the actual license plate. Since license plate dimensions are fixed depending on the geographical region, values of both *f* and $h_{o}$ are known. Thus, we only need to find out $h_{i}$, which is the height of the object of interest in the image, to be able to estimate the distance between the two vehicles. The term $h_{i}$ can be calculated in metric units as follows:

(4) $$h_{i}=k\cdot h_{ipx}$$

where $h_{ipx}$ is measured in pixels, and corresponds to the height of the object of interest or the license plate as it appears in the digital image. While *k* is a constant that has to be multiplied to $h_{ipx}$ to determine the height of the object of interest within the image in metric units.

The value of the constant *k*, also known as the pixel size, can be computed for an image of resolution M pixels wide and N pixels high, as shown:

(5) $$k=\frac{h_{s}}{N}$$

In Equation (5), the values of N and $h_{s}$ (camera sensor height) are known. Note that although the physical size of a pixel (*k*) can also be measured depending on width of the camera sensor and the digital image, since we have begun this entire analysis relying on parameters like height of the actual object and height of the object of interest in the image, thus for the same of uniformity we use the height information of the camera sensor for estimating the constant *k*.

Once pixel size (*k*) is known, we immediately replace the obtained value from Equation (5) in Equation (4):

(6) $$h_{i}=\frac{h_{s}\cdot h_{ipx}}{N}$$

The Equation (6) allows us to compute in metric units, the height of the object of interest in the image ($h_{i}$), or in other words, the height of the license plate appearing in the image. Now that $h_{i}$ is known, its value from Equation (6) is substituted in Equation (3). As a result, we have:

(7) $$∴d_{o}=f\left(1+\frac{h_{o}\cdot N}{h_{s}\cdot h_{ipx}}\right)$$

Finally, Equation (7) is the one we use in our smartphone application to estimate the distance between two vehicles from images captured by one of them.

### 3.3. Warning Generation

**Figure 5:**
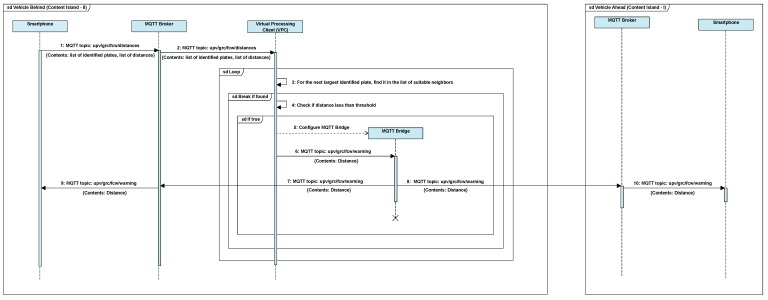
Sequence diagram of warning generation once distance estimation is complete.

Once the distance estimation is complete, a sorted list of identified plates and their corresponding distance from the camera is prepared. This information is published on the broker with topic: *upv/grc/fcw/distances*. The VPC of the island core is subscribed to this topic and receives the results obtained in the distance estimation step. These results are compared to entries in the list of suitable neighbours with the identified license plate as key. Since the application has been designed to be used in scenarios with two-lane roads with one lane per direction of traffic, and the vehicles appearing in the image captured by the back camera of a dashboard-mounted smartphone are located ahead of the current vehicle, coupled with the fact that the list of suitable neighbours consists of vehicles travelling in the same direction, we are able to identify the vehicle travelling directly ahead. Now, if the distance between the vehicle behind and the current vehicle is below the safe distance, the VPC configures the MQTT bridge using the IP information from the list of suitable neighbours. The MQTT bridge allows inter-island communication. Next, VPC publishes a warning message with topic *upv/grc/fcw/warning*, since the devices running the application are subscribed to the topic, both devices, the one located in the content island behind and the other in the content island ahead, receive the warning message. Upon receiving the warning message, a notification is displayed on screen by the device. This way, the driver of the vehicle behind, as well as the driver of the vehicle travelling ahead, are notified of the situation. All of the above steps have been presented as a sequence diagram in Figure 5.

Now, coming back to the predefined safe distance threshold, we first consider the use of the two-second rule. This rule is suggested by many government agencies, like the Road Safety Authority (RSA) [49] of Ireland, and the Department of Motor Vehicles [50] of the State of New York. According to this rule, the minimum safe distance between two vehicles travelling in the same direction depends on the velocity of the vehicle behind, and it is equal to the distance that the trailing vehicle would cover maintaining the same speed for two seconds. This way, the driver of the car behind would have enough time to react if the vehicle ahead comes to an abrupt stop. As mentioned, the application has been designed for use in urban traffic scenarios where overtaking is less common than highway scenarios. Speed limits in urban areas usually vary between 40 and 60 kmph in most countries. Considering such speed limits, according to the two-second rule, the minimum safe distance should be between 22 and 33 m. Generating warnings at such large distances would be distracting for the driver and render our application impractical, owing to the fact that such large distances between vehicles are usually not maintained in urban traffic. A more reasonable safe distance could be the length of an average vehicle. This way, we can make sure that the driver leaves enough space between his or her vehicle and the one travelling ahead, so that another vehicle could fit into that space. Since most family-sized cars are within 5 m of length, we select this distance as the minimum safe distance in our application.

## 4. Results

For this preliminary version of the application, we have studied its performance in scenarios without mobility. Photographs of static vehicles were acquired from a parking area using an Android device, which were later processed by the device to estimate the distance from the lens of the camera to the license plate to validate the proposed method. Later, the same dataset was used to measure the time required to estimate the distance by taking advantage of our edge computing solution, and compare the observed delay with a cloud-based approach.

### 4.1. Validation of the Methodology

The experiments discussed in this section were conducted to check the functionality of our approach. To perform this study, we used a Motorola Moto G-3 smartphone, which is an inexpensive Android device for daily use. It possessed a 1.4 GHz quad-core processor, 2 GB Random Access Memory (RAM), and a 13 MP main camera. For establishing the ground truth to which the calculated distance would be compared, the photographs were taken such that the distance between the lens of the camera and the license plate varied between 3 and 6 m. The actual distance was measured with a laser distance meter. The physical dimension of the license plate in the region where the experiment was conducted measured 520 by 110 mm.

First, we wanted to investigate whether the height or width of the license plate and the camera sensor should be preferred to perform distance calculation. For this purpose, we acquired images of resolutions High Definition (HD), Video Graphics Array (VGA), and Quarter Video Graphics Array (QVGA) without the use of zoom, depicting vehicles at a fixed know distance from the camera. The actual distance between the camera and the license plate was measured using a laser distance meter. Later, these images were processed and the distance was calculated using Equation (7).

**Figure 6:**
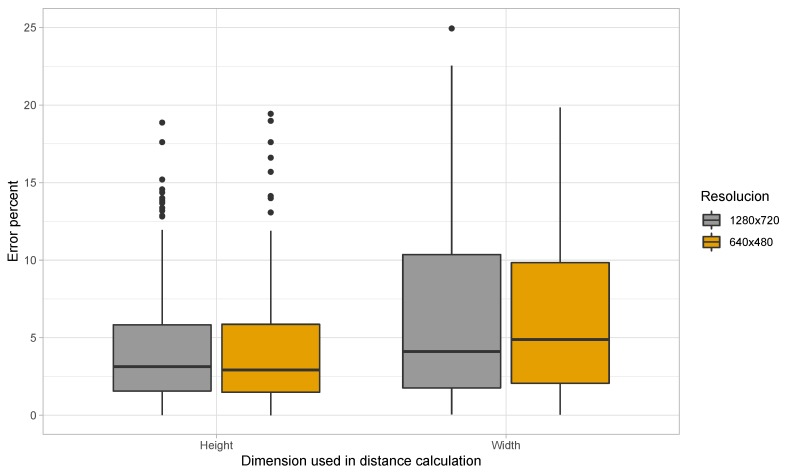
Error in distance calculation when the dimension used is varied.

Figure 6 shows the percent error in distance estimation when the dimension used in the calculation is varied. Note that, in the two different sets of experiments presented in the figure, the variation regarding whether height or width is to be used in the calculation was kept uniform across the different parameters of Equation (7); this means that, when using the height to determine the distance, we have used the height of the actual plate, the height of the digital image, the height of the camera sensor, and the height of the plate (in pixels) as it appears in the digital image for working out the distance. It can be seen from the figure that we were unable to recognise license plates with images of resolution QVGA. Furthermore, the average percent error for images of resolution HD rises from 4.25 to 4.67 when the dimension used in the calculation is switched from height to width. Under similar circumstances, the average percent error changes from 5.96 to 6.08 in case of VGA resolution. When further investigating why there was difference in the percent error depending on the dimension used in the estimation process, it was noticed that inaccuracies in the identified plate region was the source of this discrepancy. The algorithm sometimes identified the initial blue area denoting the country in EU license plates as one of the edges of the license plate. Thus, the plate region was identified to be smaller than it actually appeared in the image.

**Figure 7:**
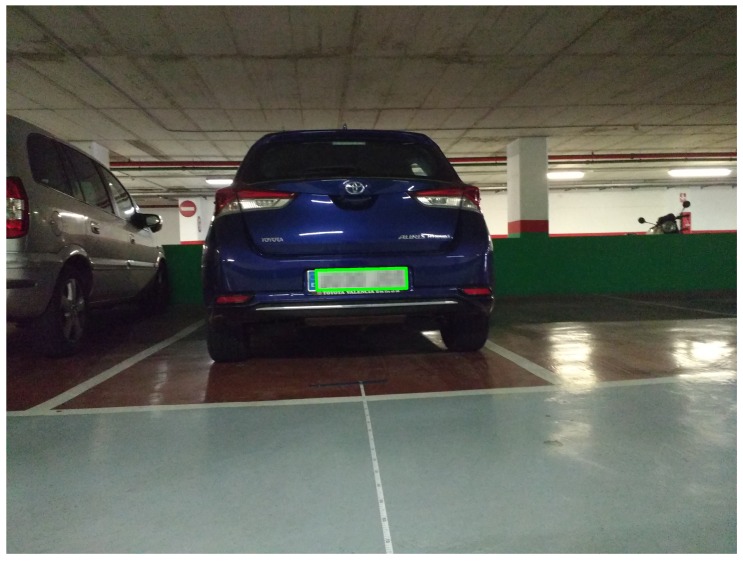
Inaccuracy in the identified plate area in one of erroneous cases.

Figure 7 depicts one such erroneous case, where the image was captured at a distance of 3 m away from the vehicle. Faulty identification of the left vertical edge of the license plate in this image can be noted. Although the license plate number was correctly identified in this case by our application, the plate in the image has been blurred for privacy reasons.

We have already established that the aim of the application is to generate warnings when two vehicles are less than 5 m apart. In the previous experiment performed we see that the detection of such distances is possible using Equation (7) with some error in the calculation. The inaccuracy may be due to human errors in the measurement of ground truth distance values, miscalculation of the identified plate region by our algorithm, and inaccurate value of the focal length of the camera supplied by the manufacturer. Also, it was seen that images with HD resolution produced lower errors in distance calculation, and that we were unable to properly identify license plates in images of resolution QVGA. Thus, from here on, we will discard the use of the lower resolution of QVGA, and focus on images of VGA and HD resolutions.

The Android Operating System (OS) allows users to specify a quality value while encoding JPEG images. The accepted value ranges from 0 to 100, where 0 is associated to the lowest quality and best compression, while the value of 100 produces images with best quality and lowest compression. It was observed that values below 20 produced images with very bad visually perceived quality, while values over 80 did not produce any significant improvement. Thus, for the next test, we acquired images of HD and VGA resolution of varying qualities to find if this quality factor of JPEG encoding affects the percent error in the estimation of distance.

**Figure 8:**
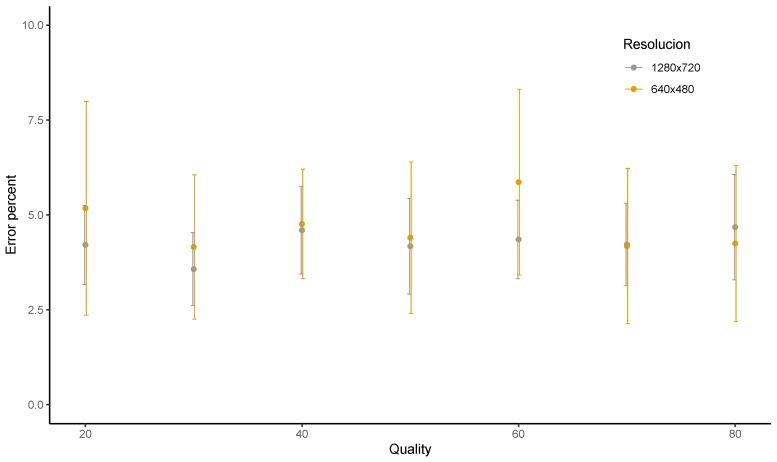
Error in distance calculation when the JPEG quality was varied.

Figure 8 summarises the observations from our experiments to study the effects of JPEG quality on the average percent error. No clear trend was observed in the graph, meaning that the percent error does not vary too much with changes in quality. When taking a closer look at the figure, we see that the average percent error for HD resolution lies between 3.58 and 4.68, with these values being achieved for qualities of 30 and 80, respectively. On the other hand, for VGA images, the average percent error varies from 4.15 to 5.86. The value of 4.15 percent was observed for a JPEG quality of 30, whereas the average percent error was 5.86 for the quality value of 60.

Thus, we conclude that the percent error in the distance calculation is not affected by the quality value of the JPEG encoding. This means that, once we are able to successfully identify the plate region and recognise it, the distance can be estimated without difficulty. Obviously, the number of successful identifications may be affected by the distance and the quality of the encoding.

### 4.2. Delay Experiments

The designed application needs to alert drivers in real time when getting too close to the vehicle ahead. Thus, apart from being accurate, it also needs to be responsive and fast in detecting such situations to generate the warnings on time. So, for the next set of experiments, we will try to find out the time taken to process the images using smartphones, and later compare the performance of our edge/fog computing solution with a cloud-based license plate recognition service.

In our next experiment, we will study the time taken by different Android devices to process and identify the license plates for various resolutions at a fixed JPEG quality of 100. Five different devices were considered for this purpose, namely a Nexus 7 tablet, a Motorola Moto G-3, a Nexus 5X, a Nexus 6, and a Samsung Galaxy Note 10.1. The Nexus 7 had a quad-core 1.2 GHz processor and 1 GB RAM. Similarly, the Moto G-3 had a 1.4 GHz quad-core processor and 2 GB RAM. The Nexus 5X was equipped with a 2 GB RAM, and an hexa-core processor with four cores running at 1.4 GHz, and the other two at 1.8 GHz. The Nexus 6 had a specification of 3 GB RAM and a 2.7 GHz quad-core processor. Finally, Note 10.1 came with a 3 GB RAM and 2.3 GHz quad-core processor.

**Figure 9:**
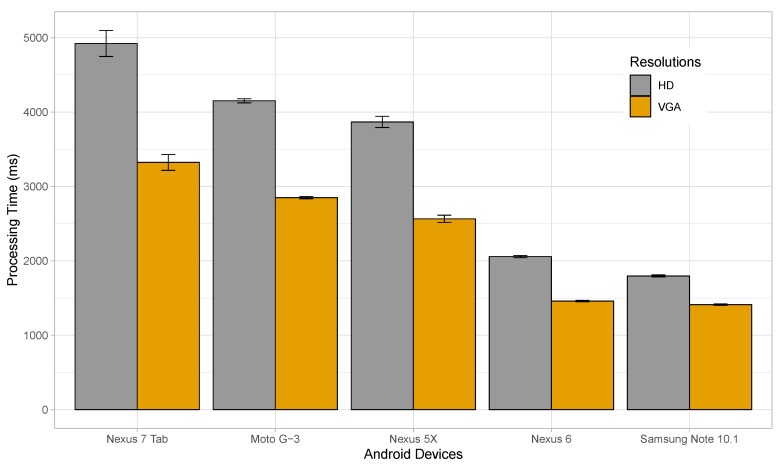
Time taken to processes images by different Android devices.

Figure 9 shows the time taken to process and identify plates in images of HD and VGA resolutions for the different devices. Only resolutions of HD and VGA were used considering the fact that images of higher resolution would only increase the processing time without providing significant advantages in license plate recognition. The time taken for processing HD images varied from 1.8 to 4.2 s, depending on the device, while it ranged from 1.4 to 3.3 s when considering VGA. Thus, lower resolution images were processed faster, and devices with faster processors performed better. The Samsung Note showed the best performance, followed by Nexus 6, Nexus 5X, Moto G-3, and Nexus 7 tablet. Note that the best processing time was achieved by the Samsung Note with 1.8 s for HD, and 1.4 s for VGA resolutions.

We have seen that the time taken to process the images by our proposed solution is a bit on the higher side, being in the order of seconds; this is due to the limited processing capacity of the smart devices used in the experiments. Better performance can be achieved with more powerful high-end devices. Also, the effect of varying JPEG quality has not been studied in the previous experiment, keeping it fixed at a maximum value. Of course, images of maximum quality are not required for the identification of the license plate. Thus, for our following experiment, we vary the JPEG quality of the images between 20 and 80, and we perform tests using the Nexus 6, one of the devices which performed better in the previous test.

**Figure 10:**
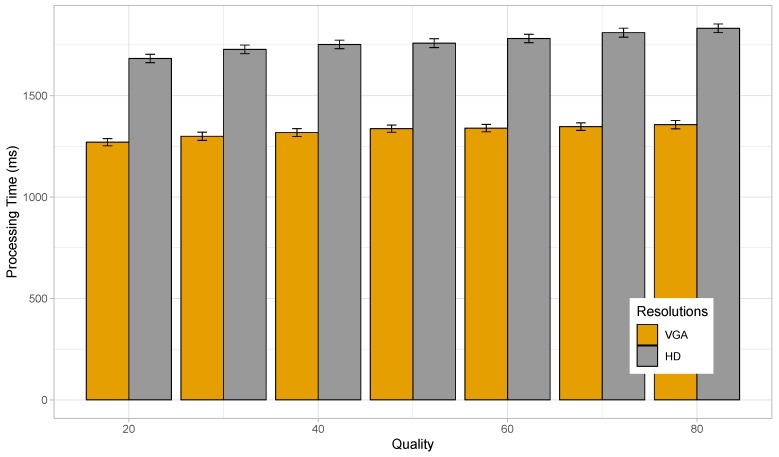
Time taken to processes images of varying quality by Nexus 6.

Figure 10 shows that, with the increase in the quality of the images, the time taken to process and identify license plates in them also increase. This is because, with the boost in quality, the image size increases and so more data needs to be processed, causing the delay involved to rise. Here, we see that, for VGA resolution, the change in JPEG quality causes the average processing time to rise from 1.270 to 1.356 s. The average processing time of 1.270 s is observed for a quality value of 20, while the highest average delay of 1.356 s occurs for a JPEG quality of 80. In case of HD resolution, the lowest average processing time of 1.683 s was detected for a JPEG quality of 20, while reaching the highest value of 1.832 s for images with a quality of 80.

Next, we wanted to compare the results obtained with a popular cloud-based license plate recognition API, called OpenALPR [51]. The OpenALPR cloud API is REpresentational State Transfer (REST)-based, and it works with virtually any programming language, and on any operating system. It analyses images of vehicles and responds with license plate data. If required, it is also capable of identifying the vehicle colour, maker, model, and body type. For our test, we have focused only on plate-related information, which is of interest to us, and results were generated much faster by the cloud API if one does not request the extra vehicle-related data. In the subsequent experiment, we employed the OpenALPR cloud API to recognise the license plates within the same set of images used in the previous tests, but focusing only on the HD resolution this time. A high-speed wired network was used to perform the analysis. The network offered high download and upload speeds of around 94.27 and 91.02 Mbps. We measured the time required to send the image over the network, the delay involved in processing the uploaded image, and the time required by the server to send us the results once the processing is complete.

**Figure 11:**
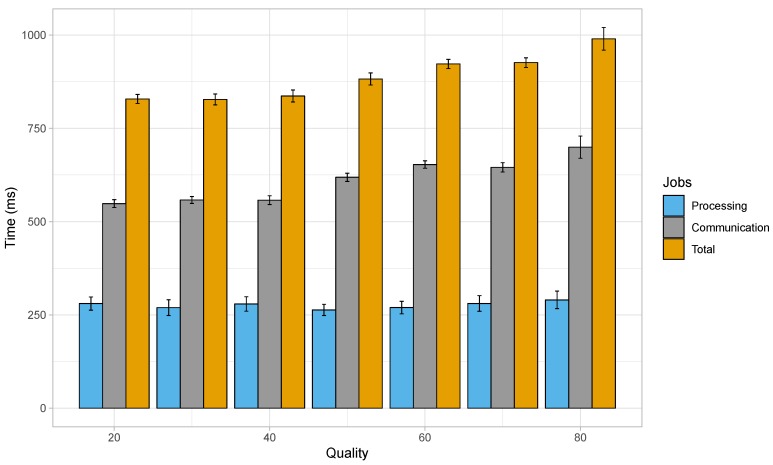
Delay when using a wired network to process images of HD resolution by a cloud server.

Figure 11 summarises the observations from the delay related experiments with the OpenALPR cloud-based service for images of HD resolution. The average processing time in this case with varying JPEG quality remained between 263 and 290 ms; these values were obtained for a JPEG quality of 50 and 80, respectively. The mean delay in the communication, that is, sending the image data and getting the response from the server, lied between 548 ms (JPEG quality of 20) and 700 ms (JPEG quality of 80). However, regarding the average total delay, which is the sum of the time taken in processing the images and the communication delay, a minimum value of 828 ms was achieved for a JPEG quality of 30, while the maximum total delay was found to be about 990 ms for a JPEG quality of 80.

The total delay involved in the upload, processing and retrieval of results when employing a cloud-based service for this purpose may apparently seem better, but it should be noted that the time taken to communicate with the remote server has a greater effect on the total delay than the time taken to identify the license plates. Also, the experimental outcome presented here has been achieved with a high speed wired connection, and such type of connectivity will not be available to networks involving vehicles in motion. Hence, in real scenarios, the total delay would be much worse due to increased communication delay.

**Figure 12:**
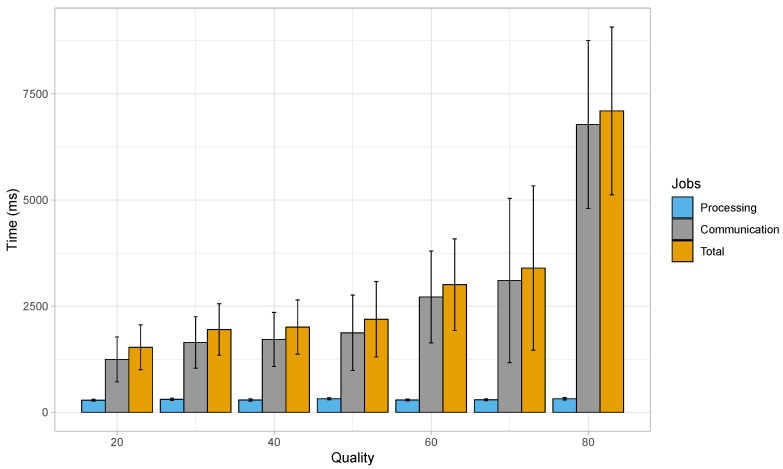
Delay when using a cellular network to process images of HD resolution by a cloud server.

Figure 12 presents the results from a similar experiment with the OpenALPR cloud-based service for images of HD resolution, but this time using a 4th Generation (4G) Long-Term Evolution (LTE) connection. This connection provided a 24.43 Mbps download, and 7.29 Mbps upload speed in a laboratory environment without involving mobility. It can be noted from this graph that the average processing time for the set of images remains within 285 and 319 ms, which is in accordance with the previous experiment involving the wired network. The lowest average processing time of 285 ms was observed for HD images of JPEG quality of 20, while the highest average processing time (319 ms) was found for HD images of JPEG quality of 50. Nevertheless, when considering the communication time, the average delay remained between 1.246 and 6.779 s, for quality values of 20 and 80, respectively. As a result, the total average delay ranges from 1.530 s (for a JPEG quality of 20) to 7.098 s (for a JPEG quality of 80). Thus, the average total delay shows a rising trend as JPEG quality increases.

If we look back at Figure 10, we can see that in our fog computing-based approach, the average processing time for HD images was of 1.683, 1.728, 1.752, 1.758, 1.781, 1.810, and 1.832 s when the JPEG quality ranged between 20 and 80 when using the Nexus 6 device. Another alternative is to depend on cloud computing approaches, where the intensive processing part would be taken care of by powerful servers, and the data to be processed is made available taking advantage of the Internet. Since our application would be adopted by smartphones present within moving vehicles, connecting to a cloud server would require the use of cellular networks in most cases. Experiments with cellular networks show that the lower data rate of such networks introduces an additional delay when compared to our proposed solution. Figure 12 showed that the total delay when using cloud-based solutions over cellular networks resulted in delays of 1.530, 1.950, 2.007, 2.191, 3.006, 3.398, and 7.098 s. Thus, we can clearly observe that, only in the case of images with HD resolution and a quality value of 20, the cloud service paired with the cellular network performed better, while for the rest of the variations in JPEG quality, our fog/edge-based approach was the clear winner. Also, since the designed application involves exchanging large amounts of data continuously, it could also increases the costs due to heavy network usage. Another important factor to be kept in mind is that cellular network coverage is not always available in very remote areas, and so this factor might hinder the application performance in such scenarios. Hence, our proposed method based on edge/fog computing, coupled with the use of more powerful smartphones to take care of the heavy processing tasks, is the better overall solution.

## 5. Conclusions

In this paper, we presented the architecture and a working prototype of a fog computing *content island* which interconnects groups of *things* packed-up together to exchange data and processing among themselves, and with other content islands. This architecture is based on MQTT, a widespread standard in IoT for publish/subscribe systems. We also presented some preliminary results regarding the prototype performance with the help of a FCW application for Android devices that takes advantage of image processing to estimate the distance between the vehicle ahead and the one following it. It estimates the distance between vehicles by identifying license plates within images captured by a dashboard-mounted Android device, owing to the fact that the physical dimension of license plates is fixed in a specific geographical region. Warnings are generated when the distance between the vehicles is less than a pre-established safe distance of 5 m. The application takes advantage of communication among various content islands to discard cases which might have caused false alarms if we just relied on the distance estimation from license plates. This is done by exchanging location information aiming to aid in the detection of which content islands share the same motion vector. The motion vector aids in identifying if a content island appearing in an image taken is travelling ahead in the same direction, coming from the opposite direction, or is simply a parked vehicle. Later, the available inter-island communication is used to alert the driver of the vehicle behind and the one travelling just ahead of it, when the established safe distance is not maintained between them.

All communications between devices present within a content island and the island core, or the communication between two islands, makes use of the MQTT protocol. The only exception to this is the communication for neighbour discovery, which is not based on MQTT. Preliminary experiments performed with our application supports our idea, and proves that distance estimation is possible at a distance of about 5 m. The error in the distance measurement was not affected by the quality of JPEG encoding, although it was observed that relying on the height parameter rather than the width for calculating the distance, provided more accuracy. Also, the total delay involved in processing images to estimate the distance was observed to be on the higher side in our proposed solution, but it is still better when compared to a cloud-based approach that relies on cellular networks for communication purposes. Also, our application would require a heavy exchange of data if it were dependent on cloud servers for processing images, and in such cases, the delay in communication using cellular networks would be greater than the time taken to analyse the images by powerful cloud servers. Relying on a cellular network for sending the image data to the cloud servers for further processing could also incur heavy network usage charges. A better solution is to adopt our proposed fog computing method using powerful high-end devices to minimise the delay involved. Thus, results achieved with our FCW application make us optimistic regarding the advantages that the proposed architecture offers: flexibility with respect to data and computation sharing, and higher fluidity in distributing in-network functions, in addition to allowing fast and scalable processing of information.

Another added advantage of our solution that relies on image processing techniques to estimate the distance between vehicles over simpler approaches that depends just on the proximity sensors available in the vehicles, is that our architecture can be easily adapted for designing other applications. In other words, while proximity sensors can aid in detecting the distance between a vehicle and an obstacle, our vision-based approach also detects and identifies the vehicle ahead; thus, it can be easily adapted to develop different sophisticated solutions. An example application which can be enhanced by embodying our solution is the overtaking assistance application [52], where a vehicle may request for real-time visual aid only from a larger vehicle located just in front that may be obstructing its view during overtaking, thus proving the utility of our proposed solution.

## Abbreviations

The following abbreviations are used in this manuscript:

4G	4th Generation (wireless/mobile communications)
API	Application Program Interface
AUTOSAR	AUTomotive Open System ARchitecture
BLOB	Binary Large OBject
CoAP	Constrained Application Protocol
FCW	Forward Collision Warning
GPS	Global Positioning System
HD	High Definition
HTTP	Hypertext Transfer Protocol
IEEE	Institute of Electrical and Electronics Engineers
IoT	Internet of Things
IP	Internet Protocol
ITS	Intelligent Transportation System
JPEG	Joint Photographic Experts Group
LBP	Local Binary Patterns
LiDAR	Light Detection and Ranging
LTE	Long-Term Evolution
MQTT	Message Queue Telemetry Transport
OBU	On-Board Unit
OS	Operating System
QVGA	Quarter Video Graphics Array
RAM	Random Access Memory
REST	REpresentational State Transfer
RSA	Road Safety Authority
RSUs	Roadside Units
SDN	Software-defined networking
UDP	User Datagram Protocol
VGA	Video Graphics Array
VNs	Vehicular Networks
VPC	Virtual Processing Client
